# The *O*-glycosylating enzyme GALNT2 acts as an oncogenic driver in non-small cell lung cancer

**DOI:** 10.1186/s11658-022-00378-w

**Published:** 2022-09-04

**Authors:** Qing Hu, Tian Tian, Yahui Leng, Yuanhui Tang, Shuang Chen, Yueyao Lv, Jingyin Liang, Yanni Liu, Tianhui Liu, Li Shen, Xiaoxia Dong

**Affiliations:** 1 Department of Clinical Oncology, Taihe Hospital, Hubei University of Medicine, 30 South Renmin Road, Shiyan, 442000 Hubei China; 2 Institute of Basic Medical Sciences, Hubei University of Medicine, Shiyan, China

**Keywords:** Non-small cell lung cancer, Oncogene, Glycosyltransferase, GALNT2

## Abstract

**Background:**

*N*-Acetylgalactosaminyltransferases (GALNTs), the enzymes that initiate mucin-type *O*-glycosylation, are closely associated with tumor occurrence and progression. However, a comprehensive analysis of GALNTs in non-small cell lung cancer (NSCLC) is lacking.

**Methods:**

The expression profiles and prognostic values of the GALNT family members in NSCLC were analyzed using publicly available databases. Gain- and loss-of-function experiments were applied to assess the biological function of GALNT2 in NSCLC. High-throughput sequencing and bioinformatics approaches were employed to uncover the regulatory mechanism of GALNT2.

**Results:**

Among the family members of GALNTs, only GALNT2 was frequently overexpressed in NSCLC tissues and was positively correlated with poor prognosis. In vitro assays showed that GALNT2 knockdown repressed NSCLC cell proliferation, migration, and invasion, but induced apoptosis and cell cycle arrest. Correspondently, GALNT2 overexpression exerted the opposite effects. In vivo experiments demonstrated that knockdown of GALNT2 restrained tumor formation in nude mice. Mechanistic investigations revealed that GALNT2 modified the *O*-glycosylation of ITGA5 and affected the activation of the PI3K/Akt and MAPK/ERK pathways. Further studies showed that miR-30d was a negative regulator of GALNT2.

**Conclusions:**

These findings suggest that GALNT2 is an oncogene in NSCLC and has the potential as a target for NSCLC therapy.

**Supplementary Information:**

The online version contains supplementary material available at 10.1186/s11658-022-00378-w.

## Background

Lung cancer ranks as the second most commonly diagnosed cancer worldwide [1]. Non-small cell lung cancer (NSCLC) is the predominant type of lung cancer. It can be classified into three histological subtypes: adenocarcinoma (LUAD), squamous cell carcinoma (LUSC), and large cell carcinoma (LCC). Although multiple therapeutic strategies have been applied to treat NSCLC, the prognosis of NSCLC patients remains dismal. Most patients usually die from tumor recurrence or metastasis. Therefore, it is of great importance to investigate the mechanism underlying NSCLC oncogenesis, which may help develop new therapeutic/preventive strategies against NSCLC.

Glycosylation is a reaction that links glycans to lipids or proteins [2]. Changes in protein glycosylation can drive the neoplastic transformation of healthy cells [3]. Aberrant glycosylation not only mediates cell–cell and cell–matrix interactions but also impacts cell growth, survival, migration, and invasion. Accumulating evidence has indicated that glycosylation alteration is a hallmark of many cancers [4–6]. Glycosylation is catalyzed by glycosyltransferases (GTs), which consist of at least 200 members. GTs are frequently dysregulated in cancers, indicating that GTs may act as oncogenes or tumor-suppressor genes [7, 8]. Thus, uncovering the relationship between GTs and NSCLC is very significant.

*N*-Acetylgalactosaminyltransferases (GALNTs) are highly eukaryotic-retaining GTs that control the initial steps of mucin-type *O*-glycosylation [9]. To date, 20 GALNT family members (from GALNT1 to GALNT20) have been identified in the human genome. GALNTs play critical roles in numerous physiological and pathological processes [10–12]. However, a comprehensive analysis of GALNTs in NSCLC has yet to be conducted. In this study, multiple online tools were utilized to evaluate the expression profiles and prognostic values of the GALNT family in NSCLC. Subsequently, in vitro and in vivo assays were carried out to clarify the biological function and regulatory mechanism of GALNT2. Our study is expected to provide a novel therapeutic target for NSCLC.

## Methods

### Data retrieving and analyzing

Differential expression and prognosis of the GALNT family members in NSCLC were analyzed using the following databases: Oncomine (http://www.oncomine.org), The Cancer Genome Atlas (TCGA, https://tcga-data.nci.nih.gov/tcga/), UALCAN (http://ualcan.path.uab.edu), Genotype-Tissue Expression (GTEx, http://commonfund.nih.gov/GTEx/), and Kaplan Meier-plotter (http://kmplot.com). The LinkedOmics database (http://www.linkedomics.org) was used to screen the genes or miRNAs associated with GALNT2 in NSCLC. The prognostic nomograms and Receiver-operating characteristic (ROC) curves were generated using the R package “rms” package or “pROC”, respectively. Gene Set Enrichment Analysis (GSEA) was performed using the GSEA software. The adjusted *P*-value < 0.05 and |log2 (fold change) | > 1 were set as the threshold.

### Microarray analysis

The cDNA microarrays of tissues (cDNA-HLugA030PG01; fifteen NSCLC samples and paired adjacent noncancerous lung tissue samples) and cells (MecDNA-HLugC042Ce01; thirteen NSCLC cell lines and one normal lung cell line) were acquired from Outdo Biotech Co., Ltd (Shanghai, China). Gene or miRNA expression analysis was performed by reverse transcription-quantitative real-time PCR (RT-qPCR).

### Cell culture, plasmids, and transfection

A549 and H1299 cell lines (Procell, Wuhan, China) were maintained in DMEM (Gibco, Carlsbad, CA, USA), supplemented with 10% fetal bovine serum (HyClone, Logan, UT, USA). GenePharma (Shanghai, China) was responsible for the synthesis of GALNT2 overexpression lentivirus (OV), GALNT2 shRNA lentivirus, and corresponding control lentiviruses (Mock or shNC). ITGA5 siRNA, negative control siRNA (siNC), miR-30d mimics, and negative control mimics were also chemically synthesized by GenePharma. Transfections were carried out using RNAi-mate reagent (GenePharma). Sequence information is provided in Additional file 1: Table S1.

### RT-qPCR

Total RNA was extracted using an RNA extraction kit (Tiangen Biotech, Beijing, China). For mRNA detection, the SuperScript IV reverse transcriptase system (Thermo Fisher Scientific, Carlsbad, CA, USA) was used for cDNA synthesis. PCR reactions were performed using the SYBR® Premix Ex Taq™ (Takara, Dalian, China). For miRNA detection, RNA was reverse transcribed into cDNA by using the miRNA First-Strand cDNA synthesis kit (Tiangen). PCR was conducted using the miRNA qPCR detection kit (Tiangen). Data were processed with the 2^−ΔΔCt^ method after normalizing to GAPDH or U6. Primer sequences are given in Additional file 1: Table S2.

### Western blot

Total protein was isolated using RIPA buffer and separated by 10% SDS-PAGE. After transferring to PVDF membranes, the blot was incubated with specific antibodies. Primary antibodies were as follows: GALNT2 (abs117502; Absin, Shanghai, China), ITGA5 (abs101364; Absin), ERK1 + ERK2 (ab184699; Abcam, Shanghai, China), p-ERK1 (T202) + p-ERK2 (T185) (ab201015; Abcam), Akt (ab8805; Abcam), p-Akt (T308) (ab38449; Abcam), and GAPDH (Absin; abs132004). Immunoreactive bands were visualized using an enhanced chemiluminescence kit (Absin).

### Cell Counting Kit-8 (CCK-8) and colony formation assays

Exponentially growing cells were seeded into 96-well plates (2 × 10^3^ cells per well). Cell viability was measured using the Cell Counting Kit-8 (CCK-8) reagent (Beyotime, Shanghai, China) according to the manufacturer’s instructions. Absorbance at 450 nm was measured. For the colony formation assay, 1 × 10^3^ cells were plated in each well of a 6-well plate. After 14 days of incubation, colonies containing more than 50 cells were stained with crystal violet.

### Cell migration and invasion assays

Transwell chambers (Corning Incorporated, Corning, NY, USA; 24-well, 8-μm pore size) uncoated or coated with Matrigel (BD Biosciences, San Jose, CA, USA) were employed to determine cell migration and invasion, respectively. A total of 5 × 10^4^ cells were seeded in the upper chamber and the complete culture medium was placed in the lower chamber. After incubation for 24 h, the migrated or invaded cells were stained with crystal violet.

### Cell cycle and apoptosis detection

Cell cycle and apoptosis were analyzed using flow cytometry as previously described by our group [13]. The cell-cycle analysis kit and Annexin V-PE/7-AAD apoptosis kit were obtained from Keygen Biotech (Nanjing, China). Operations were carried out according to the kit instructions.

### Mouse xenograft experiments

Female BALB/c nude mice, 4–5 weeks old, were purchased from the Animal Center of Hubei University of Medicine. Cells (2 × 10^6^) were injected subcutaneously into the flanks of nude mice (*n* = 5 per group). Tumor volumes were calculated using the formula: length × width^2^ × 0.5. On day 28, the mice were sacrificed. The tumors were excised and stained with Ki-67 (abs123999, Absin) as previously described by other researchers [14].

### Lectin pull-down assay

The membrane protein was extracted using a specific extraction kit (Beyotime) according to the manufacturer’s protocols. Then, Agarose bound Vicia Villosa Lectin (VVA; AL-1233-2, Vector Labs, Burlingame, CA, USA) was added to capture the lectin/glycoprotein complexes. The eluted fractions were subjected to SDS-PAGE and analyzed by Western blot.

### RNA-sequencing (RNA-Seq)

RNA-seq was conducted by Biotecan Pharmaceuticals Co., Ltd (Shanghai, China). Total RNA from A549 cells with or without GALNT2 knockdown was subjected to RNA-seq. Differentially expressed genes (DEGs) were identified using the DESeq2 R package. The screening criterion was set as false discovery rate (FDR) < 0.05 and | log2 (fold change)| ≥ 1. DEGs were subjected to GO functional enrichment (Gene Ontology, http://www.geneontology.org/) and KEGG pathway analysis (Kyoto Encyclopedia of Genes and Genomes, http://www.genome.jp/kegg/).

### Luciferase assay

The wild-type and mutant 3′-untranslated region (3′-UTR) of human GALNT2 were constructed and cloned into the pGL3 luciferase reporter vector (GenePharma). Subsequently, the above two vectors were co-transfected with miR-30d mimics or negative control mimics into A549 and H1299 cells, respectively. After 48 h of transfection, cells were collected for the luciferase activity assay using a Dual-Luciferase Reporter Assay System (Promega, Madison, WI, USA).

### Statistical analysis

Data are presented as mean ± SD. Statistical analysis was done using the GraphPad Prism software (San Diego, CA, USA). Statistical significance was determined by Student’s *t*-test, chi-square test, ANOVA, or Pearson test. *P* < 0.05 was considered statistically significant.

## Results

### A comprehensive analysis of the GALNT family in NSCLC

**Fig. 1:**
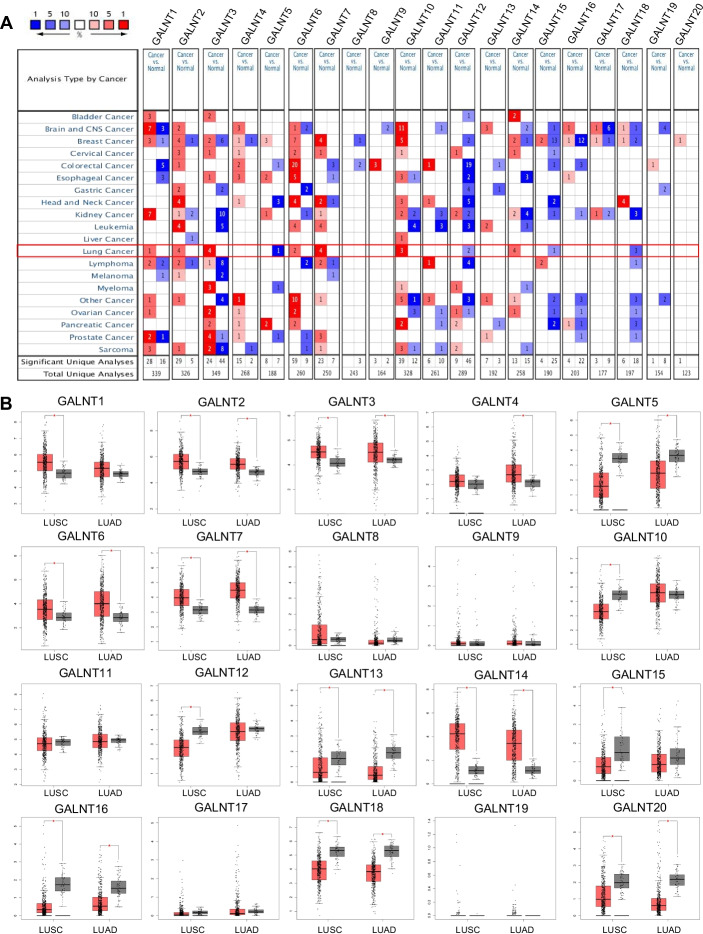
Gene expression profiles of the GALNT family members in NSCLC. **A** Oncomine analysis of GALNT mRNA expression. Red: up-regulation; blue: down-regulation. The number in the colored cell represents the number of datasets meeting the threshold (*P*-value, 0.05; fold change, 2; gene rank, top 10%). **B** Analysis of GALNT mRNA expression in TCGA. Red box represents tumor tissues and grey box represents normal tissues. *P < 0.05

To define the expression patterns of GALNTs in NSCLC, the mRNA levels of 20 GALNTs in NSCLC (LUAD, LUSC, and LCC) tissues and adjacent normal lung tissues were analyzed using the Oncomine database (Fig. 1A and Additional file 1: Table S3). Compared with the normal lung tissues, the mRNA levels of GALNT2/3/6/7/10/14 were elevated in LUAD tissues, and GALNT15/18 mRNA levels were reduced. Moreover, GALNT1/2/3/14 mRNA levels were higher, while GALNT12/18 mRNA levels were lower in LUSC tissues than in normal lung tissues. Additionally, the mRNA levels of GALNT2/14 were increased, but GALNT5/12 mRNA levels were decreased in LCC tissues compared with normal lung tissues. Subsequently, the mRNA levels of 20 GALNTs in NSCLC (mainly LUAD and LUSC) tissues were investigated based on the integrated analysis of TCGA and GTEx datasets (Fig. 1B). We observed that GALNT2/3/6/7/14 mRNA levels were upregulated in NSCLC tissues compared with the normal lung tissues; by contrast, GALNT5/13/16/18/20 mRNA levels were downregulated. Global gene expression analysis indicated that GALNT2 and GALNT14 were highly expressed, whereas GALNT5 was lowly expressed in NSCLC.

**Fig. 2:**
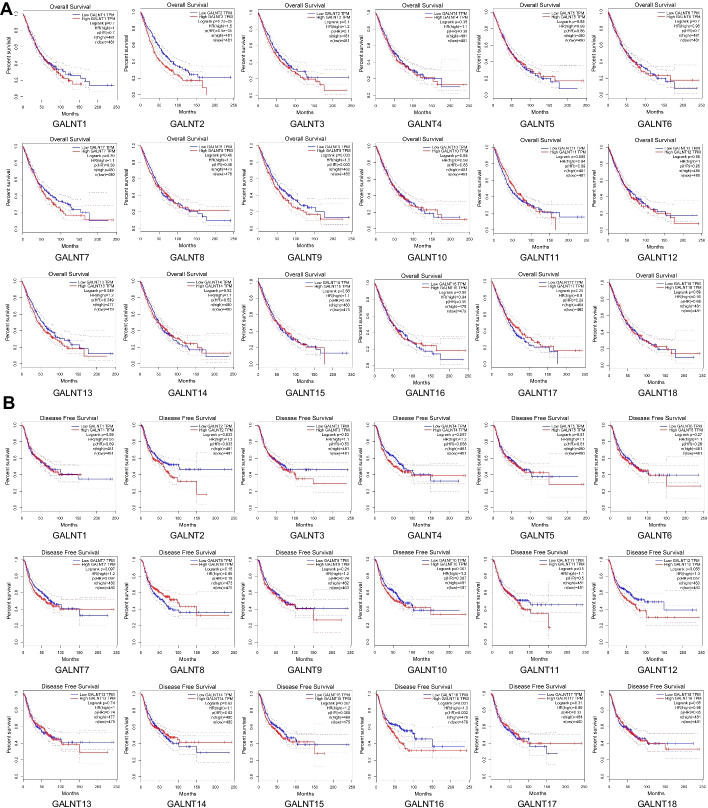
Prognostic values of the GALNT family members in NSCLC. **A** Relationship between GALNT expression and overall survival. **B** Relationship between GALNT expression and disease free survival. Data for GALNT19 and GALNT20 were not available

To determine the prognostic values of GALNTs in NSCLC, Kaplan–Meier survival analysis was performed. We found that high expression of GALNT2/9/13 was linked to decreased overall survival (Fig. 2A). Besides, High GALNT2/16 expression was associated with unfavorable disease-free survival (Fig. 2B). TCGA data analysis showed that only GALNT2 was related to the poor prognosis of all NSCLC patients. Furthermore, the survival information extracted from the Kaplan–Meier plotter database also confirmed that GALNT2 expression was negatively correlated with the overall survival of NSCLC patients (Additional file 1: Fig. S1). Considered collectively, we selected GALNT2 as a candidate gene for further study.

### Clinical importance of GALNT2 in NSCLC

**Fig. 3:**
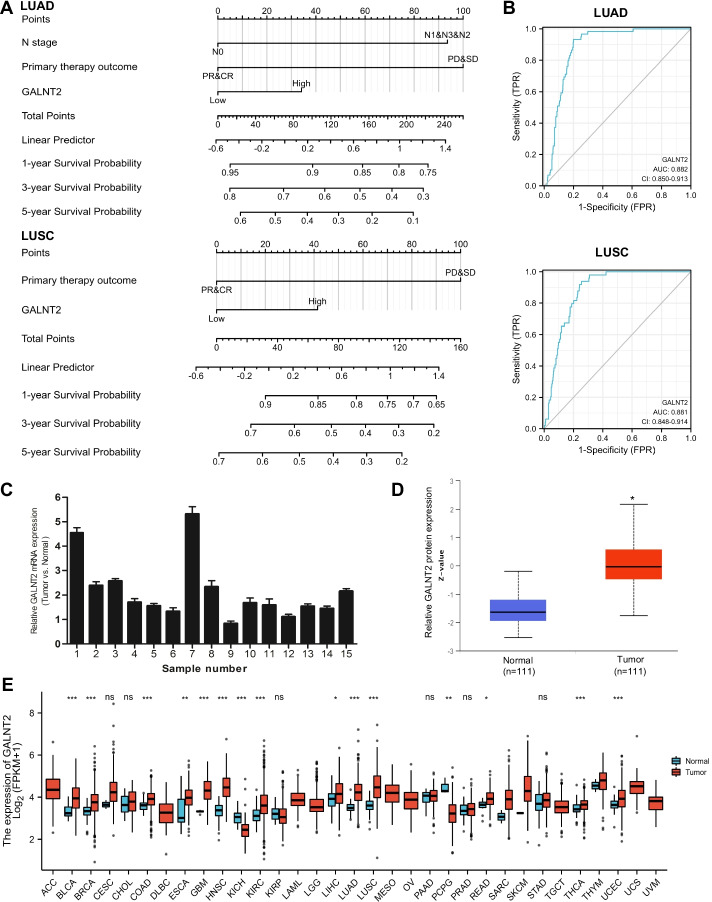
Clinical significance of GALNT2 in NSCLC. **A** Nomogram predicting overall survival probability. **B** ROC curves. **C** Detection of GALNT2 expression by RT-qPCR in NSCLC tissues. **D** Analysis of GALNT2 protein expression in NSCLC tissues by UALCAN. **E** TCGA pan-cancer analysis of GALNT2. NS, no significance; *P < 0.05; **P < 0.01; ***P < 0.001

RNA-Seq data for NSCLC were downloaded from the TCGA database. Univariate analysis showed that T stage, M stage, pathologic stage, primary therapy outcome, and GALNT2 expression were significantly associated with the overall survival. Multivariate Cox regression hazard analysis revealed that GALNT2 was an independent prognostic factor for overall survival (Additional file 1: Tables S4 and S5). A nomogram was established to predict the probability of the 1-, 3- and 5-year overall survival of NSCLC patients by integrating all the independent prognostic indicators (Fig. 3A). The ROC curve indicated that GALNT2 expression could serve as a promising biomarker for distinguishing NSCLC patients from normal controls (Fig. 3B). To verify the expression pattern of GALNT2 in NSCLC, RT-qPCR was performed. We confirmed that GALNT2 mRNA was overexpressed in NSCLC specimens (Fig. 3C). The results from the UALCAN website also demonstrated that GALNT2 protein expression was upregulated in NSCLC samples (Fig. 3D). To characterize GALNT2 abundance in human cancers, we examined GALNT2 expression using the TCGA pan-cancer data. We discovered that the expression of GALNT2 was widely elevated across numerous cancers (Fig. 3E). These findings suggested that GALNT2 might play a key role as an oncogene in NSCLC.

### Oncogenic role of GALNT2 in NSCLC

**Fig. 4:**
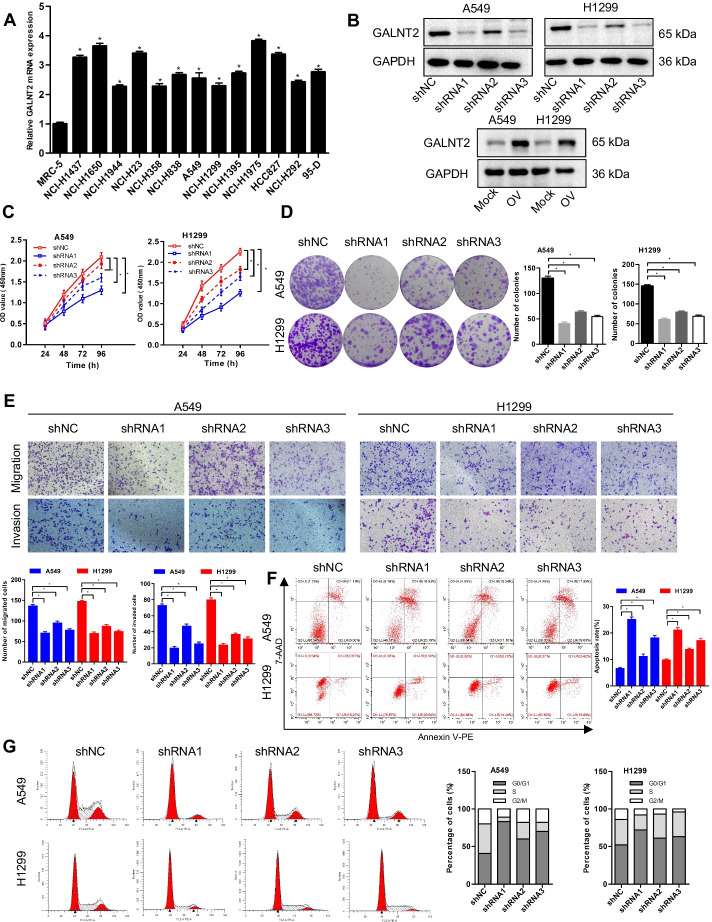
GALNT2 knockdown inhibits the malignant properties of NSCLC cells. **A** Detection of GALNT2 expression by RT-qPCR. **B** Detection of GALNT2 expression by Western blot. **C** Cell proliferation assay. **D** Colony formation assay. **E** Transwell migration and Matrigel invasion assays (magnification, ×100). **F** Flow cytometric analysis of cell cycle. **G** Analysis of cell apoptosis by flow cytometry. *OV* GALNT2 overexpression lentivirus, *Mock* normal control lentivirus, *shRNA* GALNT2 shRNA lentivirus, *shNC* negative control shRNA lentivirus. *P < 0.05

**Fig. 5:**
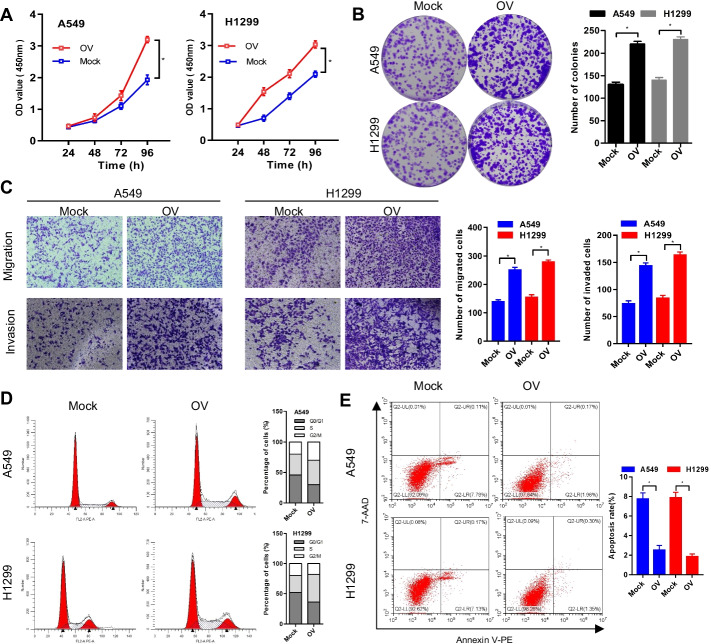
GALNT2 overexpression promotes malignant phenotypes of NSCLC cells. **A** CCK-8 assay. **B** Colony formation assay. **C** Transwell migration and Matrigel invasion assays (magnification, ×100). **D** Flow cytometric analysis of cell cycle. **E** Analysis of cell apoptosis by flow cytometry. *OV* GALNT2 overexpression lentivirus, *Mock* normal control lentivirus. *P < 0.05

To elucidate the biological function of GALNT2 in NSCLC, we analyzed the expression of GALNT2 in a panel of NSCLC cell lines and a normal lung cell line using RT-qPCR. Upregulation of GALNT2 expression was frequently detected in various NSCLC cell lines (Fig. 4A). Then, we conducted loss- and gain-of-function assays to explore whether GALNT2 could affect the malignant behaviors of NSCLC cells. Successful overexpression or knockdown of GALNT2 in A549 and H1299 cells was confirmed by RT-qPCR (Additional file 1: Fig. S2) and Western blot (Fig. 4B). The CCK-8, colony formation, and Transwell assays demonstrated that GALNT2 knockdown repressed the proliferative, migratory, and invasive abilities of NSCLC cells (Fig. 4C–E). Flow cytometry analysis revealed that knockdown of GALNT2 promoted apoptosis and induced G0/G1 cell cycle arrest (Fig. 4F, G). Overexpression of GALNT2 in A549 and H1299 cells, however, had the opposite effects. GALNT2 overexpression stimulated cell proliferation, migration, invasion, and cell cycle progression, and inhibited apoptosis (Fig. 5A–E). Together, these functional studies illustrated that GALNT2 exerted a tumor-promoting property in NSCLC.

### *GALNT2 facilitates NSCLC cell growth *in vivo

**Fig. 6:**
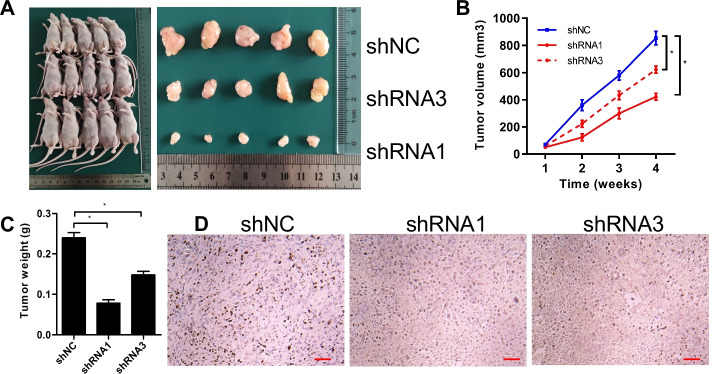
GALNT2 knockdown suppresses tumor growth in vivo. **A** Representative images of xenograft tumors formed. **B** Tumor growth curves. **C** Tumor weights. **D** Ki67 staining of tumor tissues (bar = 50 µm). *shRNA* GALNT2 shRNA lentivirus, *shNC* negative control shRNA lentivirus. *P < 0.05

To check whether GALNT2 could regulate tumorigenesis in vivo, A549 cells were subcutaneously injected into nude mice. Tumor volumes were measured every week. We discovered that the sizes and weights of tumors in the GALNT2 knockdown group were smaller than those in the control group; thus, knockdown of GALNT2 suppressed tumor formation in vivo (Fig. 6A–C). Meanwhile, the expression of Ki67, a marker of cell proliferation, was decreased in the GALNT2 knockdown group (Fig. 6D). These results confirmed the oncogenic potential of GALNT2 in NSCLC.

### ITGA5 is a target glycoprotein of GALNT2 in NSCLC

**Fig. 7:**
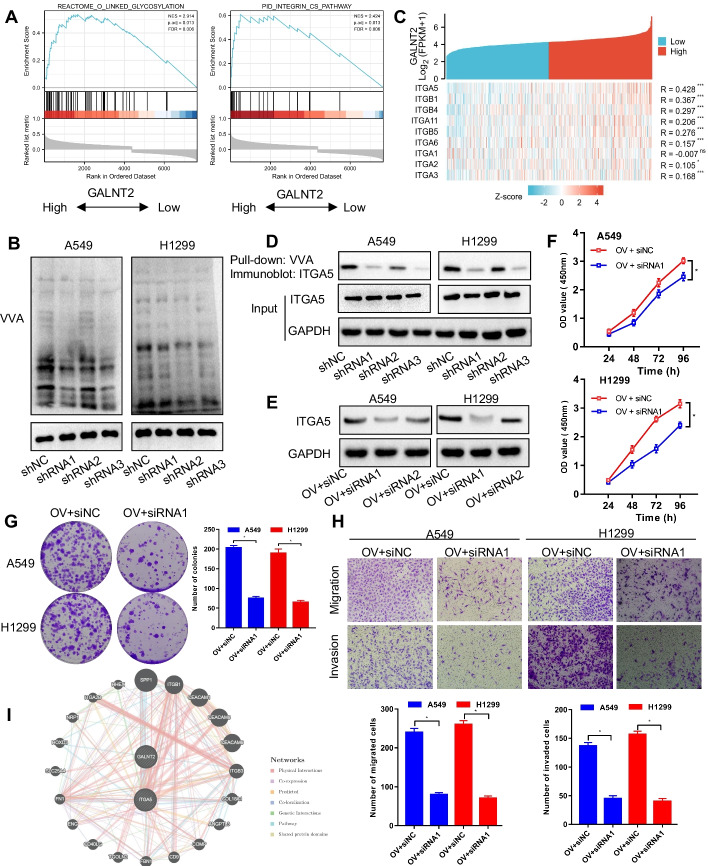
ITGA5 can be *O*-glycosylated by GALNT2 in NSCLC. **A** Functional pathway analysis by GSEA. **B** VVA binding assay for the detection of *O*-linked glycosylation. **C** Heatmap displaying the Pearson correlation between GALNT2 and the Integrin pathway-related genes. **D** Detection of ITGA5 *O*-glycosylation by pull-down assay. **E** Western blot analysis of ITGA5 expression following siRNA transfection. **F** CCK-8 assay. **G** Colony formation assay. **H** Transwell migration and Matrigel invasion assays (magnification, ×100). **I** Protein–protein interaction network. *OV* GALNT2 overexpression lentivirus, *Mock* normal control lentivirus, *shRNA* GALNT2 shRNA lentivirus, *shNC* negative control shRNA lentivirus, *siRNA* ITGA5 siRNA, *siNC* negative control siRNA, *NS* no significance; *P < 0.05; **P < 0.01; ***P < 0.001

To uncover the regulatory mechanism of GALNT2 in NSCLC, we used LinkedOmics to screen the genes co-expressed with GALNT2. These genes were uploaded into GSEA for enrichment analysis (Additional file 2: Table S6). We observed a positive association between GALNT2 expression and the *O*-linked glycosylation or Integrin pathway (Fig. 7A). Lectin blot analysis proved that *O*-glycosylation was suppressed following GALNT2 knockdown, as shown by reduced VVA staining in NSCLC cells (Fig. 7B). Among genes identified in the LinkedOmics database as related to the Integrin pathway, ITGA5 was most strongly correlated with GALNT2 (Fig. 7C). To evaluate whether GALNT2 could modify ITGA5 *O*-glycosylation, a lectin pull-down assay was performed. Interestingly, ITGA5 was pulled down by VVA, and the knockdown of GALNT2 decreased VVA binding to ITGA5 (Fig. 7D). To assess whether ITGA5 was implicated in GALNT2-mediated malignant phenotypes of NSCLC cells, we silenced ITGA5 expression with siRNA in GALNT2-overexpressing cells (Fig. 7E). We found that GALNT2-induced cell proliferation, migration, and invasion were attenuated by silencing ITGA5 (Fig. 7F–H). Moreover, the interaction between GALNT2 and ITGA5 was validated by GeneMANIA (http://genemania.org/) (Fig. 7I). Thus, ITGA5 acted as a downstream effector of GALNT2 in NSCLC.

### GALNT2 can activate the PI3K/Akt and MAPK/ERK pathways in NSCLC

**Fig. 8:**
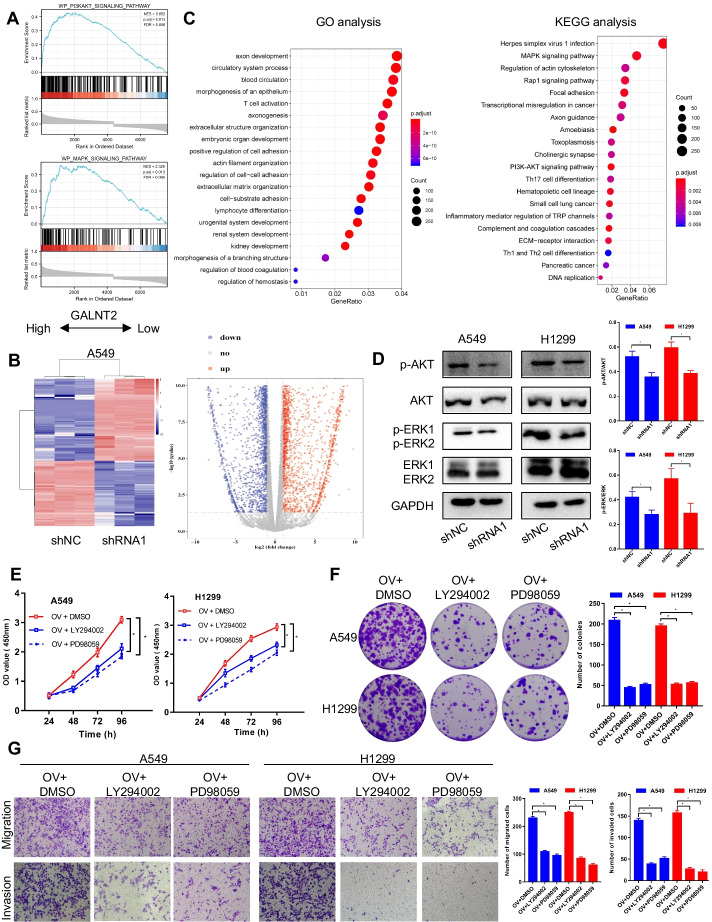
The PI3K/Akt and MAPK/ERK pathways can be regulated by GALNT2 in NSCLC. **A** Enrichment plots from GSEA. **B** Heatmap and volcano plot of DEGs. **C** GO annotation and KEGG analysis of DEGs. **D** Effects of GALNT2 knockdown on the expression of PI3K/AKT and MAPK/ERK pathways. **E** CCK-8 assay. **F** Colony formation assay. **G** Transwell migration and Matrigel invasion assays (magnification, ×100). *OV* GALNT2 overexpression lentivirus, *shRNA* GALNT2 shRNA lentivirus, *shNC* negative control shRNA lentivirus. *P < 0.05

GSEA analysis implied a crucial role for GALNT2 in the regulation of PI3K/Akt and MAPK pathways (Fig. 8A; Additional file 2: Table S6). To verify the GSEA results, we knocked down GALNT2 in A549 cells and extracted RNA for RNA-seq analysis (Additional file 3: Table S7). Different gene expression profiles were displayed between GALNT2-knockdown and control cells (Fig. 8B). DEGs were subjected to GO and KEGG for annotation. KEGG analysis revealed that DEGs were also enriched in the PI3K/Akt and MAPK pathways (Fig. 8C). To validate the association between these two signaling pathways and GALNT2, the total protein and phosphorylation levels of Akt and ERK were examined via Western blot. As expected, the phosphorylation levels of Akt and ERK, but not the total levels of Akt and ERK, were reduced following GALNT2 knockdown (Fig. 8D). Furthermore, LY294002 (PI3K/Akt inhibitor, 10 μmol/L) or PD98059 (MAPK/ERK inhibitor, 10 μmol/L) could attenuate the promotive effects of GALNT2 on NSCLC cell proliferation, migration, and invasion (Fig. 8E–G). Therefore, the pro-oncogenic role of GALNT2 in NSCLC was related to the activation of the PI3K/Akt and MAPK/ERK pathways.

### miR-30d is an upstream regulator of GALNT2 in NSCLC

**Fig. 9:**
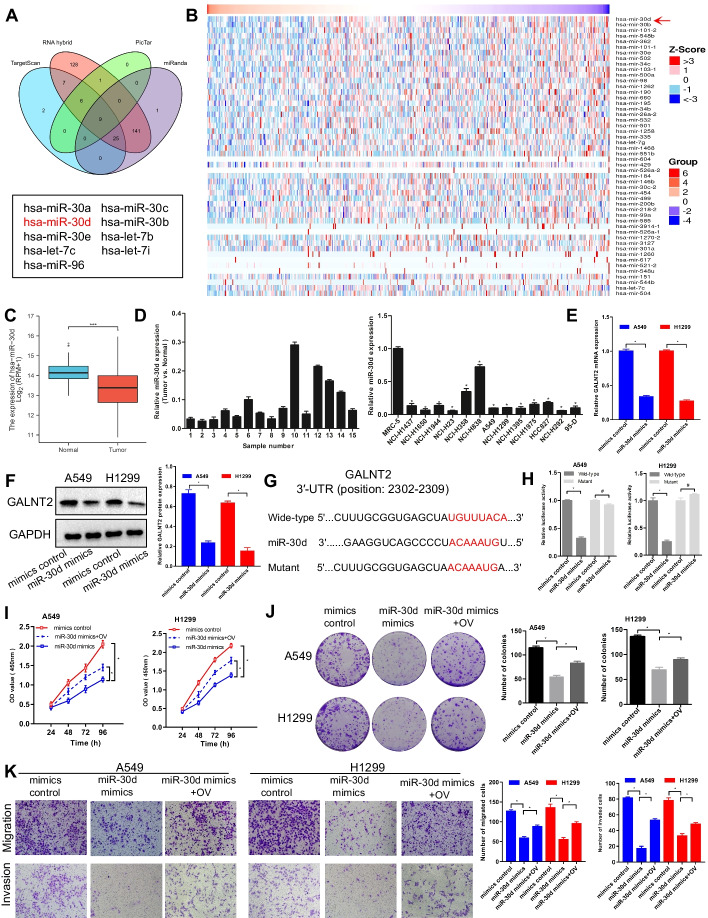
miR-30d negatively regulates GALNT2 expression in NSCLC. **A** Four databases were employed to predict the potential miRNAs targeting GALNT2. **B** Heatmaps of the top 50 miRNAs negatively associated with GALNT2 in NSCLC. **C** Analysis of miR-30d expression in TCGA. **D** Analysis of miR-30d expression using cDNA microarrays. **E** Detection of GALNT2 expression by RT-qPCR. **F** Detection of GALNT2 expression by Western blot. **G** Schematic representation of the putative binding site for miR-30d in the GALNT2 3′-UTR. **H** Luciferase reporter assay. **I** CCK-8 assay. **J** Colony formation assay. **K** Transwell migration and Matrigel invasion assays (magnification, ×100). *OV* GALNT2 overexpression lentivirus. ^#^*P* > 0.05, *P < 0.05

**Fig. 10:**
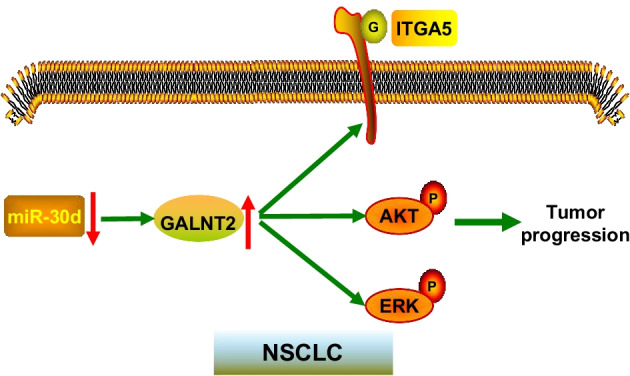
A proposed model for the regulatory mechanism of GALNT2. G: *O*-glycosylated; P: phosphorylated

To explore the mechanism underlying GALNT2 upregulation in NSCLC, the miRNAs potentially targeting GALNT2 were predicted using online analysis tools, such as TargetScan, RNA hybrid, PicTar, and miRanda. We found 9 potential miRNAs, which could interact with GALNT2 (Fig. 9A). In addition, the top 50 miRNAs associated with GALNT2 in NSCLC were screened out by the Pearson correlation test via LinkedOmics (Fig. 9B). After taking the intersection of those predicted miRNAs, we discovered that miR-30d presented the strongest association with GALNT2. Data from TCGA and our microarrays indicated that miR-30d was downregulated in NSCLC tissues and cell lines (Fig. 9C, D). Consequently, miR-30d was selected as the candidate miRNA. To further investigate whether GALNT2 was regulated by miR-30d, RT-qPCR and Western blot assays were conducted. Remarkably, both mRNA and protein levels of GALNT2 in A549 and H1299 cells were inhibited by miR-30d (Fig. 9E, F). Subsequently, the potential binding sequence between GALNT2 and miR-30d was predicted using TargetScan (Fig. 9G). As anticipated, miR-30d reduced the luciferase activity of the wild-type GALNT2 3′-UTR, while the luciferase activity of the mutant GALNT2 3′-UTR was not changed (Fig. 9H). To determine how the miR-30d/GALNT2 axis affected the malignant properties of NSCLC cells, rescue assays were performed. We discovered that miR-30d suppressed NCSCL cell proliferation, migration, and invasion, but these inhibition effects were restored by GALNT2 overexpression (Fig. 9I–K). All the above information pointed out that miR-30d was a negative modulator of GALNT2 in NSCLC. A schematic diagram was generated to illustrate the working mechanism of GALNT2 in NSCLC (Fig. 10).

## Discussion

Altered glycosylation is a universal feature of human cancers. Glycosylation alteration also frequently occurs in lung cancer [15, 16]. Mucin-type *O*-glycosylation, which is produced by the GALNT family, is a fundamental form of glycosylation. GALNTs are ubiquitously expressed in a variety of tissues, and some GALNTs have oncogenic or tumor-suppressive properties. In the present study, we identified GALNT2, among the GALNT family members, as a key oncogene in NSCLC. This study is the first to clarify the critical role of GALNT2 in NSCLC. These findings enrich our understanding of the pathogenesis of NSCLC.

Multiple lines of evidence demonstrated that GALNT2 was dysregulated in several solid tumors, such as glioma [17], neuroblastoma [18], gastric adenocarcinoma [19], oral squamous cell carcinoma [20], and hepatocellular carcinoma [21]. Several recent independent studies revealed that GALNT2 was overexpressed in lung adenocarcinoma [22, 23]. The present study, however, showed that GALNT2 was upregulated in all subtypes of NSCLC. High expression of GALNT2 was closely correlated with the poor prognosis in NSCLC patients. Both loss- and gain-of-function experiments proved that GALNT2 was indispensable for NSCLC cell proliferation, migration, and invasion. In addition, GALNT2 was able to regulate cell cycle progression and apoptosis. Moreover, GALNT2 exerted pro-tumorigenic activity in nude mice. Thus, our study provides evidence that GALNT2 plays an oncogenic role in NSCLC.

Numerous proteins could serve as substrates for the GALNT family. For example, the *O*-glycosylation of SARS-CoV-2 spike protein was mediated by GALNT1 [24]. Loss of GALNT3 in pancreatic cancer was related to aberrant *O*-glycosylation of the ErbB family [25]. EGFR *O*-glycosylation in breast cancer was impacted by GALNT8 [11]. It was suggested that GALNT2 modulated the *O*-glycosylation of EGFR in glioma [17]. We confirmed that ITGA5 was *O*-glycosylated by GALNT2 in NSCLC, indicating that the substrates of GALNT2 may differ in various tissues. ITGA5, also known as integrin α5, is an important member of the integrin family. Integrins are transmembrane glycoproteins that participate in a range of biological events, including proliferation, adhesion, migration, and invasion [26]. Integrins have been extensively documented to play essential roles in NSCLC progression [27–29]. Importantly, the glycosylation of integrins is necessary for their proper folding and functionality [30]. In the current study, we found that the knockdown of GALNT2 inhibited ITGA5 *O*-glycosylation. The promotive effects of GALNT2 on the malignant phenotypes of NSCLC cells were reversed by ITGA5 silencing. Hence, we have reason to believe that ITGA5 acts as a major downstream effector of GALNT2 in NSCLC.

The PI3K/Akt and MAPK/ERK pathways are involved in cell proliferation, migration, invasion, and survival [31, 32]. The two pathways are commonly activated in NSCLC [33, 34]. Indeed, our GSEA and RNA-Seq data followed by functional assays confirmed that the oncogenic potential of GALNT2 in NSCLC was linked to the activation of the PI3K/Akt and MAPK/ERK pathways. The findings of our study provide new insights into the mechanism by which GALNT2 contributes to NSCLC tumorigenesis and malignant progression.

It is generally known that miRNAs can down-regulate the transcription of target genes [35]. However, it is not yet clear which miRNAs regulate the abundance of GALNT2 in NSLLC. It was found that GALNT2 expression was negatively modulated by let-7b in IgA nephropathy [36]. We discovered that miR-30d targeted GALNT2 and suppressed its expression in NSCLC. It has previously been suggested that miR-30d is a well-characterized tumor-suppressive miRNA [37]. To date, there is no published research on the association between miR-30d and GALNT2. In this study, we confirmed that miR-30d repressed NCSCL cell proliferation, migration, and invasion, whereas the inhibitory effects of miR-30d were partially abrogated by GALNT2 overexpression. Therefore, miR-30d is an important upstream regulator of GALNT2 in NSCLC.

Undoubtedly, there are some limitations to this study. GALNT2 protein expression was not examined in NSCLC tissues. The *O*-glycosylation sites of ITGA5 are not well understood. It is of note that there exist other *O*-glycosylated proteins, which are not explored in this study. Further investigations are required to construct the regulatory network of miRNAs in controlling GALNT2 expression.

## Conclusions

To sum up, we systematically illustrated the expression profile, prognostic value, biological function, and regulatory mechanism of GALNT2 in NSCLC. GALNT2 overexpression was frequently detected in NSCLC and could serve as a prognosis predictor. High expression of GALNT2 exhibited a tumor-promoting function in NSCLC. GALNT2 was able to enhance ITGA5 *O*-glycosylation and activate the PI3K/Akt and MAPK/ERK pathways. Additionally, the expression of GALNT2 was negatively regulated by miR-30d. As an important oncogene, GALNT2 has emerged as a potential therapeutic target for NSCLC.

## Supplementary Information

**Additional file 1: Table S1.** The sequences of miRNAs, siRNAs, and shRNAs. **Table S2.** Primer sequences used for RT-qPCR. **Table S3.** Transcriptional expression patterns of the GALNT family members in different lung cancer datasets (Oncomine database). **Table S4.** Univariate and multivariate Cox regression analysis in the TCGA-LUAD cohort. **Table S5.** Univariate and multivariate Cox regression analysis in the TCGA-LUSC cohort. **Figure S1.** The relationship between GALNT2 expression and overall survival in NSCLC. The data was retrieved from the Kaplan–Meier plotter database. **Figure S2.** The expression of GALNT2 was detected by RT-qPCR. *P < 0.05; **P < 0.01.

**Additional file 2: Table S6.** GESA enrichment analysis of genes co-expressed with GALNT6 in NSCLC.

**Additional file 3: Table S7.** RNA-seq after GALNT2 knockdown in A549 cells.

## Data Availability

All data generated or analyzed during this study are included in this published article and its Additional files.

## Abbreviations

GALNTs: *N*-Acetylgalactosaminyltransferases
GTs: Glycosyltransferases
NSCLC: Non-small cell lung cancer
TCGA: The Cancer Genome Atlas
GSEA: Gene Set Enrichment Analysis
RT-qPCR: Transcription-quantitative real-time PCR
CCK-8: Cell Counting Kit-8
DEGs: Differentially expressed genes
VVA: Vicia Villosa Lectin
