# Supplementary material for: The O-glycosylating enzyme GALNT2 acts as an oncogenic driver in non-small cell lung cancer
Source: Cell Mol Biol Lett. 2022 Sep 4;27:71. doi: 10.1186/s11658-022-00378-w (PMC9440866; doi:10.1186/s11658-022-00378-w)

**Table S1.** The sequences of miRNAs, siRNAs, and shRNAs

| **Name** | **Sequences (5**′**-3**′**)** |
| --- | --- |
| GALNT2 shRNA1 | GGTGATCACGTTTCACAATGA |
| GALNT2 shRNA2 | GGATGACTACAGCAATGATCC |
| GALNT2 shRNA3 | GGAAGTACGACATGATGATGG |
| Negative control shRNA | TTCTCCGAACGTGTCACGT |
| ITGA5 siRNA1 | CCCUCUGACAUUGAUUGUUTT  AACAAUCAAUGUCAGAGGGTT |
| ITGA5 siRNA2 | CCACAUUGGUUACCACUAATT  UUAGUGGUAACCAAUGUGGTT |
| Negative control siRNA | UUCUCCGAACGUGUCACGUTT  ACGUGACACGUUCGGAGAATT |
| miR-30d mimics | UGUAAACAUCCCCGACUGGAAG  UCCAGUCGGGGAUGUUUACAUU |
| Negative control mimics | UUCUCCGAACGUGUCACGUTT  ACGUGACACGUUCGGAGAATT |

**Table S2.** Primer sequences used for RT-qPCR

| **Name** |  | **Sequences (5′-3′)** |
| --- | --- | --- |
| GALNT2 | Forward  Reverse | CTGGGGAGTGGTAGAAGCAA  AGACACTTGCGAATGGAGGA |
| miR-30d | Forward  Reverse | CCTGTTGGTGCACTTCCTAC  TGCAGTAGTTCTCCAGCTGC |
| U6 | Forward  Reverse | ATGACGTCTGCCTTGGAGAAC  TCAGTGTGCTACGGAGTTCAG |
| GAPDH | Forward  Reverse | TGAACGGGAAGCTCACTGG  TCCACCACCCTGTTGCTGTA |

**Table S3. Transcriptional expression patterns of the GALNT family members in different lung cancer datasets (Oncomine database)**

| **GALNTs** | **Comparisons** | **Fold change** | **P-value** | **Dataset** |
| --- | --- | --- | --- | --- |
| GALNT1 | LUSC vs. Normal | 3.024 | 4.33E-4 | Bhattacharjee Lung |
| GALNT2 | LCC vs. Normal  LUAD vs. Normal  LUSC vs. Normal  LUSC vs. Normal | 3.817  3.388  3.074  2.324 | 0.002  8.10E-4  9.62E-4  0.005 | Garber Lung  Garber Lung  Garber Lung Bhattacharjee Lung |
| GALNT3 | LUAD vs. Normal  LUSC vs. Normal  LUAD vs. Normal  LUAD vs. Normal | 3.715  5.890  2.315  2.529 | 1.32E-4  2.52E-5  2.80E-14  8.05E-10 | Bhattacharjee Lung  Bhattacharjee Lung  Okayama Lung  Selamat Lung |
| GALNT4 | NA | NA | NA | NA |
| GALNT5 | LCC vs. Normal | -4.420 | 1.07E-26 | Hou Lung |
| GALNT6 | LUAD vs. Normal  LUAD vs. Normal | 2.147  2.244 | 3.57E-14  1.08E-11 | Selamat Lung  Okayama Lung |
| GALNT7 | LUAD vs. Normal  LUAD vs. Normal  LUAD vs. Normal  LUAD vs. Normal | 2.943  2.795  2.465  2.062 | 3.25E-38  2.15E-20  1.19E-8  9.48E-10 | Okayama Lung  Landi Lung  Su Lung  Hou Lung |
| GALNT8 | NA | NA | NA | NA |
| GALNT9 | NA | NA | NA | NA |
| GALNT10 | LUAD vs. Normal  LUAD vs. Normal  LUAD vs. Normal | 2.067  2.633  2.921 | 1.07E-30  0.003  1.10E-6 | Okayama Lung  Bhattacharjee Lung  Su Lung |
| GALNT11 | NA | NA | NA | NA |
| GALNT12 | LUSC vs. Normal  LCC vs. Normal | -2.853  -5.069 | 0.001  1.47E-7 | Garber Lung  Hou Lung |
| GALNT13 | NA | NA | NA | NA |
| GALNT14 | LUAD vs. Normal  LCC vs. Normal  LUAD vs. Normal  LUSC vs. Norma | 3.327  4.628  2.812  3.770 | 1.23E-5  7.95E-7  1.12E-9  9.00E-9 | Su Lung  Hou Lung  Hou Lung  Hou Lung |
| GALNT15 | LUAD vs. Normal | -2.148 | 2.02E-10 | Okayama Lung |
| GALNT16 | NA | NA | NA | NA |
| GALNT17 | NA | NA | NA | NA |
| GALNT18 | LUAD vs. Normal  LUSC vs. Normal  LUAD vs. Normal | -2.939  -2.357  -2.066 | 2.92E-15  8.98E-14  1.18E-15 | Hou Lung  Hou Lung  Selamat Lung |
| GALNT19 | NA | NA | NA | NA |
| GALNT20 | NA | NA | NA | NA |

NA: not available.

**Table S4.** Univariate and multivariate Cox regression analysis in the TCGA-LUAD cohort

| **Characteristics** | **Total(N)** | **Univariate analysis** | | **Multivariate analysis** | |
| --- | --- | --- | --- | --- | --- |
|  |  | **Hazard ratio**  **(95% CI)** | **P-value** | **Hazard ratio**  **(95% CI)** | **P-value** |
| T stage | 523 |  |  |  |  |
| T1&T2 | 457 | Reference |  |  |  |
| T3&T4 | 66 | 2.317 (1.591-3.375) | <0.001 | 1.665 (0.909-3.051) | 0.099 |
| N stage | 510 |  |  |  |  |
| N0 | 343 | Reference |  |  |  |
| N1&N2&N3 | 167 | 2.601 (1.944-3.480) | <0.001 | 1.854 (1.192-2.883) | 0.006 |
| M stage | 377 |  |  |  |  |
| M0 | 352 | Reference |  |  |  |
| M1 | 25 | 2.136 (1.248-3.653) | 0.006 | 1.514 (0.625-3.665) | 0.358 |
| Age | 516 |  |  |  |  |
| <=65 | 255 | Reference |  |  |  |
| >65 | 261 | 1.223 (0.916-1.635) | 0.172 |  |  |
| Gender | 526 |  |  |  |  |
| Female | 280 | Reference |  |  |  |
| Male | 246 | 1.070 (0.803-1.426) | 0.642 |  |  |
| Pathologic stage | 518 |  |  |  |  |
| Stage I&Stage II | 411 | Reference |  |  |  |
| Stage III&Stage IV | 107 | 2.664 (1.960-3.621) | <0.001 | 1.313 (0.718-2.402) | 0.376 |
| GALNT2 | 526 |  |  |  |  |
| Low | 264 | Reference |  |  |  |
| High | 262 | 1.486 (1.114-1.983) | 0.007 | 1.334 (0.891-1.999) | 0.034 |
| Primary therapy outcome | 439 |  |  |  |  |
| PD&SD | 108 | Reference |  |  |  |
| PR&CR | 331 | 0.377 (0.268-0.530) | <0.001 | 0.393 (0.260-0.594) | <0.001 |

**Table S5.** Univariate and multivariate Cox regression analysis in the TCGA-LUSC cohort

| **Characteristics** | **Total(N)** | **Univariate analysis** | | **Multivariate analysis** | |
| --- | --- | --- | --- | --- | --- |
|  |  | **Hazard ratio**  **(95% CI)** | **P-value** | **Hazard ratio**  **(95% CI)** | **P-value** |
| T stage | 496 |  |  |  |  |
| T1&T2 | 403 | Reference |  |  |  |
| T3&T4 | 93 | 1.658 (1.200-2.291) | 0.002 | 1.689 (0.984-2.896) | 0.057 |
| N stage | 490 |  |  |  |  |
| N0 | 317 | Reference |  |  |  |
| N1&N2&N3 | 173 | 1.151 (0.869-1.523) | 0.327 |  |  |
| M stage | 415 |  |  |  |  |
| M0 | 408 | Reference |  |  |  |
| M1 | 7 | 3.112 (1.272-7.616) | 0.013 | 2.642 (0.943-7.403) | 0.064 |
| Gender | 496 |  |  |  |  |
| Female | 130 | Reference |  |  |  |
| Male | 366 | 1.211 (0.879-1.669) | 0.241 |  |  |
| Age | 490 |  |  |  |  |
| <=65 | 190 | Reference |  |  |  |
| >65 | 300 | 1.279 (0.960-1.704) | 0.093 |  |  |
| Pathologic stage | 492 |  |  |  |  |
| Stage I&Stage II | 402 | Reference |  |  |  |
| Stage III&Stage IV | 90 | 1.570 (1.139-2.163) | 0.006 | 0.941 (0.528-1.679) | 0.838 |
| GALNT2 | 496 |  |  |  |  |
| Low | 246 | Reference |  |  |  |
| High | 250 | 1.537 (1.170-2.018) | 0.002 | 1.569 (1.059-2.323) | 0.025 |
| Primary therapy outcome | 357 |  |  |  |  |
| PD&SD | 47 | Reference |  |  |  |
| PR&CR | 310 | 0.281 (0.186-0.422) | <0.001 | 0.256 (0.152-0.431) | <0.001 |

**Figure S1.** The relationship between GALNT2 expression and overall survival in NSCLC. The data was retrieved from the Kaplan-Meier plotter database.


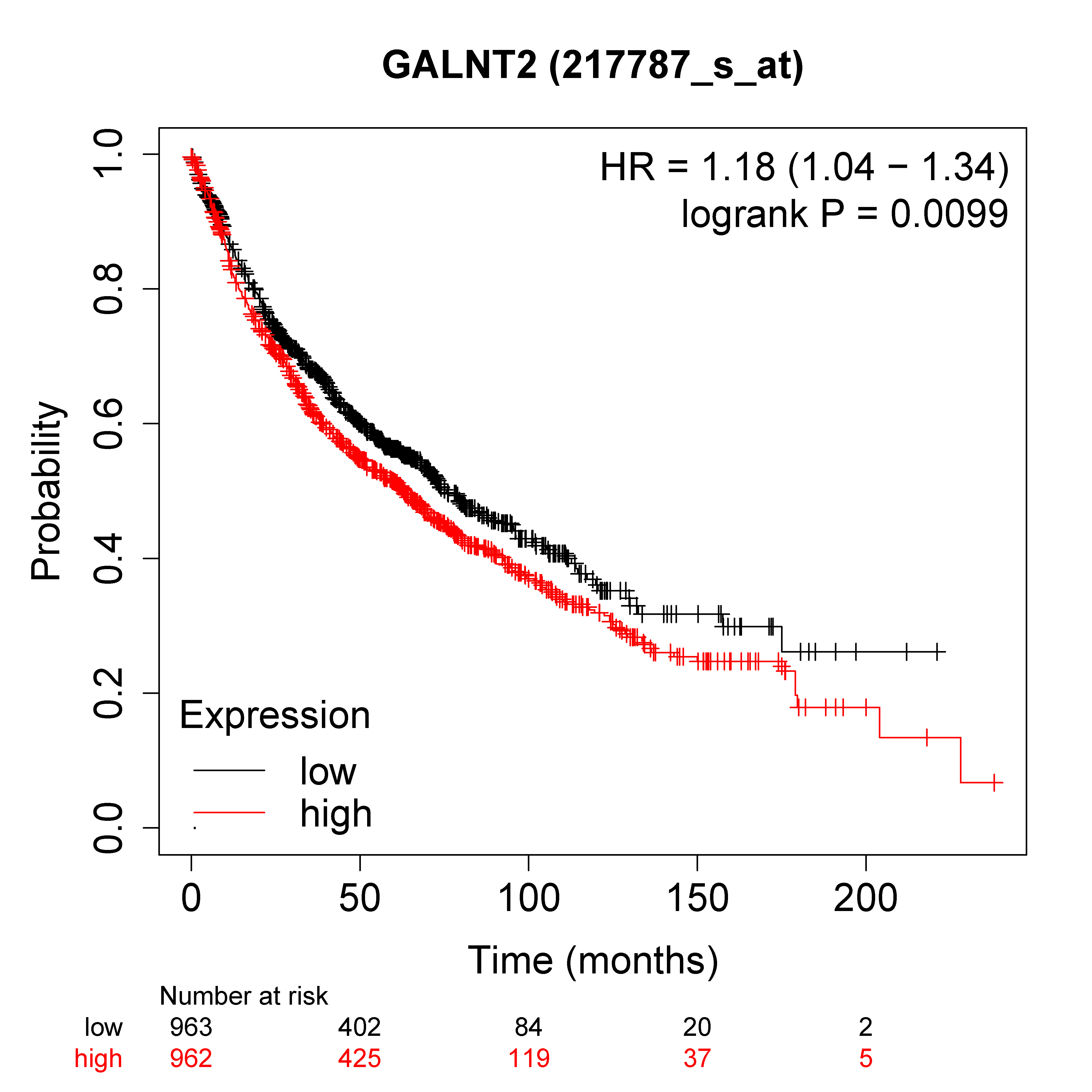


**Figure S2.** The expression of GALNT2 was detected by RT-qPCR. ^*^P < 0.05; ^**^P < 0.01.


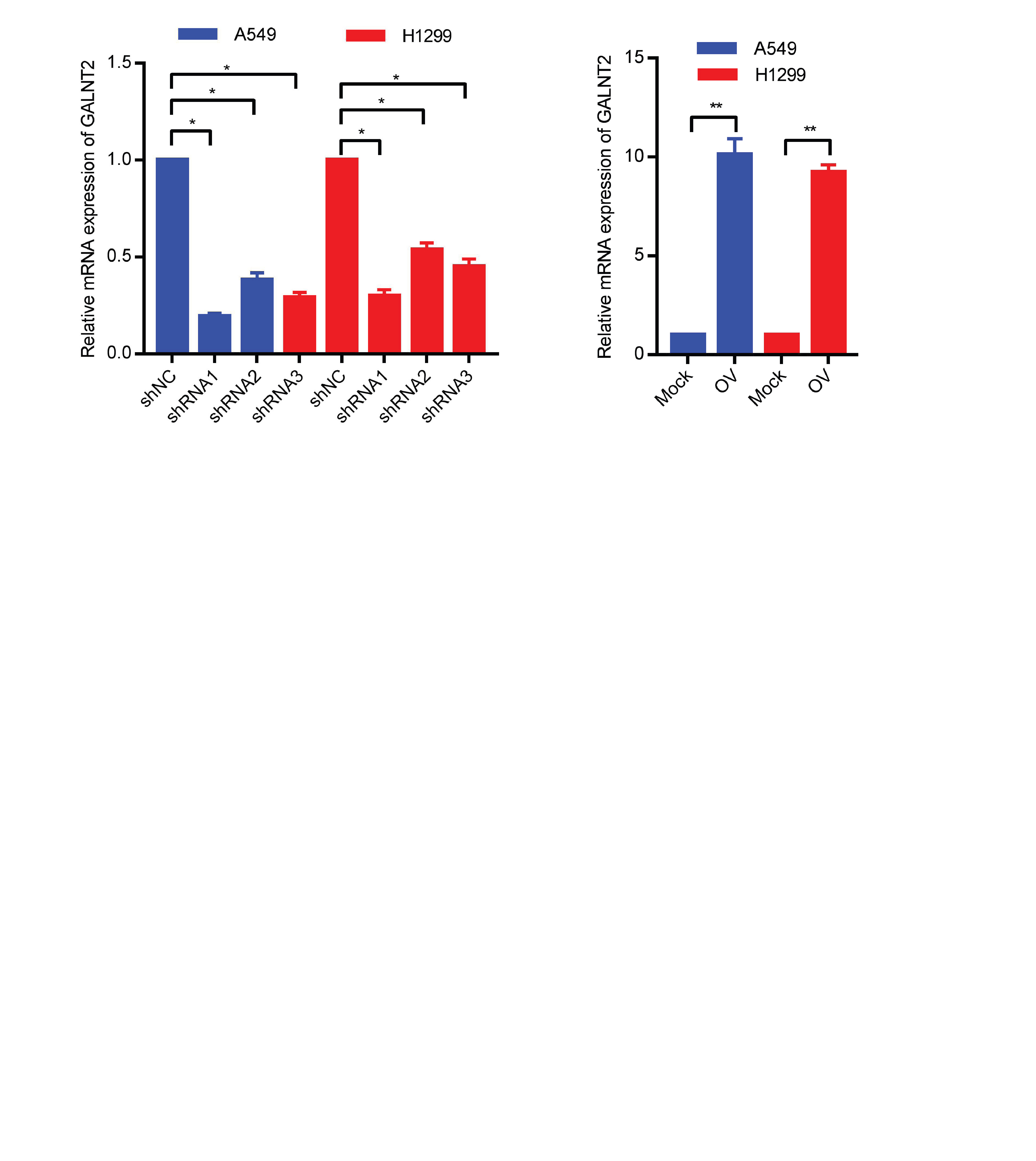

Supplement: Supplementary file 1 — Additional file 1: Table S1. The sequences of miRNAs, siRNAs, and shRNAs. Table S2. Primer sequences used for RT-qPCR. Table S3. Transcriptional expression patterns of the GALNT family members in different lung cancer datasets (Oncomine database). Table S4. Univariate and multivariate Cox regression analysis in the TCGA-LUAD cohort. Table S5. Univariate and multivariate Cox regression analysis in the TCGA-LUSC cohort. Figure S1. The relationship between GALNT2 expression and overall survival in NSCLC. The data was retrieved from the Kaplan–Meier plotter database. Figure S2. The expression of GALNT2 was detected by RT-qPCR. *P < 0.05; **P < 0.01. [file 11658_2022_378_MOESM1_ESM.doc]
